# Small non-coding RNA landscape is modified by GPAT2 silencing in MDA-MB-231 cells

**DOI:** 10.18632/oncotarget.25582

**Published:** 2018-06-15

**Authors:** Ezequiel Lacunza, Mauro Aldo Montanaro, Annamaria Salvati, Domenico Memoli, Francesca Rizzo, Maria Florencia Henning, Ivana Yoseli Quiroga, Hervé Guillou, Martín Carlos Abba, María del Rosario Gonzalez-Baro, Alessandro Weisz, Magalí Pellon-Maison

**Affiliations:** ^1^ Centro de Investigaciones Inmunológicas Básicas y Aplicadas, Facultad de Ciencias Médicas, Universidad Nacional de La Plata, La Plata, Argentina; ^2^ Instituto de Investigaciones Bioquímicas de La Plata, Consejo Nacional de Investigaciones Científicas y Técnicas (CONICET), Universidad Nacional de La Plata, La Plata, Argentina; ^3^ Laboratory of Molecular Medicine and Genomics, Department of Medicine, Surgery and Dentistry “Scuola Medica Salernitana”, University of Salerno, Baronissi, SA, Italy; ^4^ Genomix4Life, Department of Medicine, Surgery and Dentistry “Schola Medica Salernitana”, University of Salerno, Baronissi, SA, Italy; ^5^ Toxalim, Université de Toulouse, INRA, ENVT, INP-Purpan, UPS, Toulouse, France

**Keywords:** GPAT2, breast cancer, piRNAs, tRNA derived fragments, small non-coding RNAs

## Abstract

Glycerol-3-phosphate acyltransferase-2 is a member of “cancer-testis gene” family. Initially linked to lipid metabolism, this gene has been recently found involved also in PIWI-interacting RNAs biogenesis in germline stem cells. To investigate its role in piRNA metabolism in cancer, the gene was silenced in MDA-MB-231 breast cancer cells and small RNA sequencing was applied. PIWI-interacting RNAs and tRNA-derived fragments expression profiles showed changes following GPAT2 silencing. Interestingly, a marked shift in length distribution for both small RNAs was detected in GPAT2-silenced cells. Most downregulated PIWI-interacting RNAs are single copy in the genome, intragenic, hosted in snoRNAs and previously found to be upregulated in cancer cells. Putative targets of these PIWI-interacting RNAs are linked to lipid metabolism. Downregulated tRNA derived fragments derived from, so-called ‘differentiation tRNAs’, whereas upregulated ones derived from proliferation-linked tRNAs. miRNA amounts decrease after Glycerol-3-phosphate acyltransferase-2 silencing and functional enrichment analysis of deregulated miRNA putative targets point to mitochondrial biogenesis, IGF1R signaling and oxidative metabolism of lipids and lipoproteins. In addition, miRNAs known to be overexpressed in breast cancer tumors with poor prognosis where found downregulated in GPAT2-silenced cells. In conclusion, GPAT2 silencing quantitatively and qualitatively affects the population of PIWI-interacting RNAs, tRNA derived fragments and miRNAs which, in combination, result in a more differentiated cancer cell phenotype.

## INTRODUCTION

Glycerol-3-phosphate acyltransferase (GPAT) catalyzes the first step in glycerolipid biosynthesis, in which glycerol-3-phosphate is acylated to form lysophosphatidic acid. In mammals, four GPAT isoforms (GPAT1–GPAT4) have been described which differ in their subcellular location, tissue expression pattern, substrate preference, transcriptional regulation, and sensitivity to sulfhydryl group reagents such as N-ethylmaleimide [1]. While GPAT1, GPAT3 and GPAT4 are expressed in lipogenic tissues and regulated by nutritional status, we found that GPAT2 is a mitochondrial isoform that is highly expressed in the testis, where its expression is transient, being restricted mainly to primary spermatocytes [2]. At subsequent stages of meiosis, GPAT2 expression is abruptly turned off by methylation of its promoter [3].

Although GPAT2 was initially associated with lipid metabolism [2] a recent work links GPAT2 to the biogenesis of Piwi-interacting RNAs (piRNAs) [4]. piRNAs are a class of small non-coding RNAs (sncRNAs) of 24-31 nt in length that function in germline cells to silence retrotransposons and maintain genome integrity [5]. piRNA expression is high at pachytene stage of meiosis, which supports a role of GPAT2 in this pathway. It has been described that GPAT2 physically interacts with MILI, a key component of piRNA pathway, and its knockdown in germline stem cells significantly reduces the amounts of piRNAs bound to MILI [4].

We also determined that GPAT2 behaves as a cancer testis gene: its expression is restricted to testis under physiological conditions, but it is ectopically overexpressed in cancer cells, contributing to tumor phenotype. GPAT2 knockdown in MDA-MB-231 breast cancer cells diminished cell proliferation, anchorage independent growth, migration and tumorigenicity, and increased staurosporine-induced apoptosis. In contrast, GPAT2 over-expression increased cell proliferation rate and resistance to staurosporine-induced apoptosis [6].

Increasing evidence indicates that non-coding RNAs (ncRNA) play a relevant role in the progression of cancer. Non-coding RNAs can be subdivided into two major classes based on their transcript size: sncRNAs (20-200 nucleotides) and long ncRNAs (lncRNAs, over 200 nucleotides). Small non-coding RNAs (sncRNA), include RNAs involved in translation such as transfer RNAs (tRNAs) and ribosomal RNAs (rRNAs), small nuclear RNAs (snRNAs) involved in splicing events, and small nucleolar RNAs (snoRNAs) involved in the modification of other small RNAs, such as rRNAs and tRNAs. Additionally, sncRNAs include RNAs with regulatory functions, such as short-interfering RNAs (siRNAs), microRNAs (miRNAs), the above mentioned piRNAs and the recently characterized tRNA derived fragments (tRF). Although miRNAs are the most extensive subclass of sncRNAs studied in cancer, the involvement of piRNAs and tRF has also been described [7, 8]. piRNAs are deregulated in cancer cells, where they could play a role in tumorigenesis [9, 10, 11, 12]. Each tumor type expresses specific subgroups of piRNAs and some of them correlate with relevant clinical parameters [8, 13]. In this sense, it has been shown that piRNA pathway is active in breast cancer cells and that specific piRNAs are differentially expressed in breast cancer respect to mammary epithelial cells [10]. Moreover, a group of piRNAs differentially expressed in exponentially growing cells compared to quiescent cells after estrogen deprivation, has been identified leading to the conclusion that piRNA expression responds to exogenous mitogenic stimuli [10].

Considering that GPAT2 has been involved in piRNA biogenesis in germline stem cells and that GPAT2 is highly expressed in MDA-MB-231 cells, we asked whether GPAT2 could be also involved in piRNA metabolism in breast cancer cells. Considering that GPAT2 knockdown reduced tumor phenotype, we hypothesized that this action could be related to changes in specific piRNAs associated with cell proliferation. To test this hypothesis, GPAT2 knockdown was performed in MDA-MB-231 cells and sncRNA expression was assessed.

## RESULTS

### GPAT2 silencing modifies the sncRNA expression pattern of breast cancer cells

**Figure 1:**
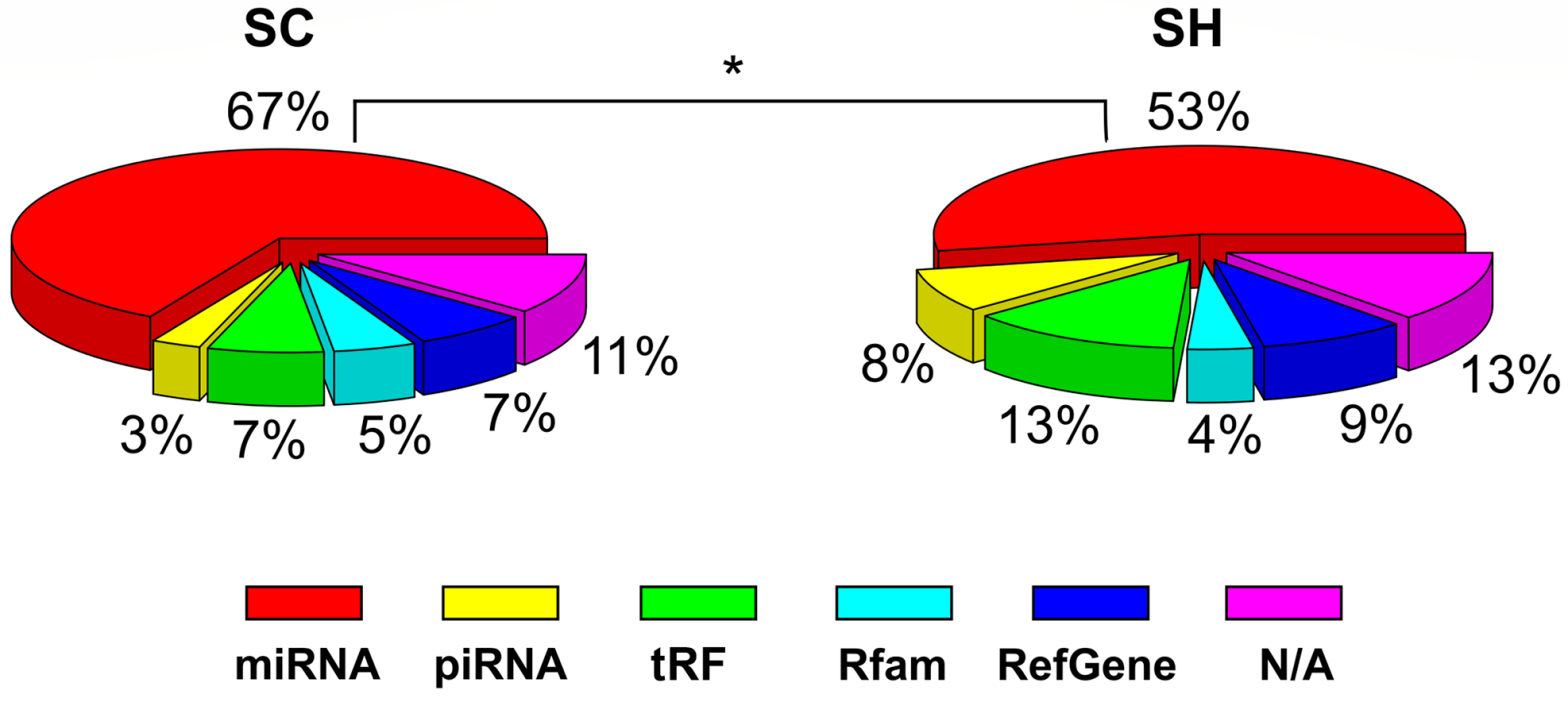
sncRNA distribution in SC and SH cells Piecharts of the percentages of aligned reads assigned to each category of sncRNA in the SC cells and SH cells. A significant decrease was observed in the abundance of miRNAs of the SH cells ^*^ p value≤0.05.

To analyze the impact of GPAT2 on piRNA biogenesis in the MDA-MB-231 cells, GPAT2 silencing was performed by shRNA plasmid transfection and puromycin selection to establish scramble control cells (SC) and GPAT2 silenced cells (SH) GPAT2 mRNA expression was reduced by 90% and GPAT2 protein was undetectable in SH cells (Supplementary Figure 1). Small RNA sequencing was performed on these cell lines. Results demonstrate that silencing GPAT2 affects sncRNA distribution. In SC cells, we identified an average of 67% of miRNAs, 3% of piRNAs, 7% of tRF, 5% of Rfam, 7% of Refgene and 11% of non-assigned transcripts, whereas in SH cells, 53% were miRNAs, 8% piRNAs, 13% tRF, 4% Rfam, 9% Refgene and 13% not-assigned transcripts (Figure 1). Although percentages of total reads for each category differed in SC and SH cells, differences were only significant for the miRNA category, with a decrease after GPAT2 silencing (Figure 1; Supplementary Table 1). In addition, we compared miRNA expression profiles between SC *vs* MDA-MB-231 and MCF10 cell lines obtained from the study of Zhou et al. [14]. As expected, we found a high correlation between SC *vs* MDA-MB-231 cells from Zhou et al., whereas no correlation was found for the comparison between SC and MCF10 cell line (Supplementary Figure 2).

### GPAT2 modulates the expression of snoRNA derived piRNAs and piRNAs related to breast cancer cell proliferation

**Figure 2:**
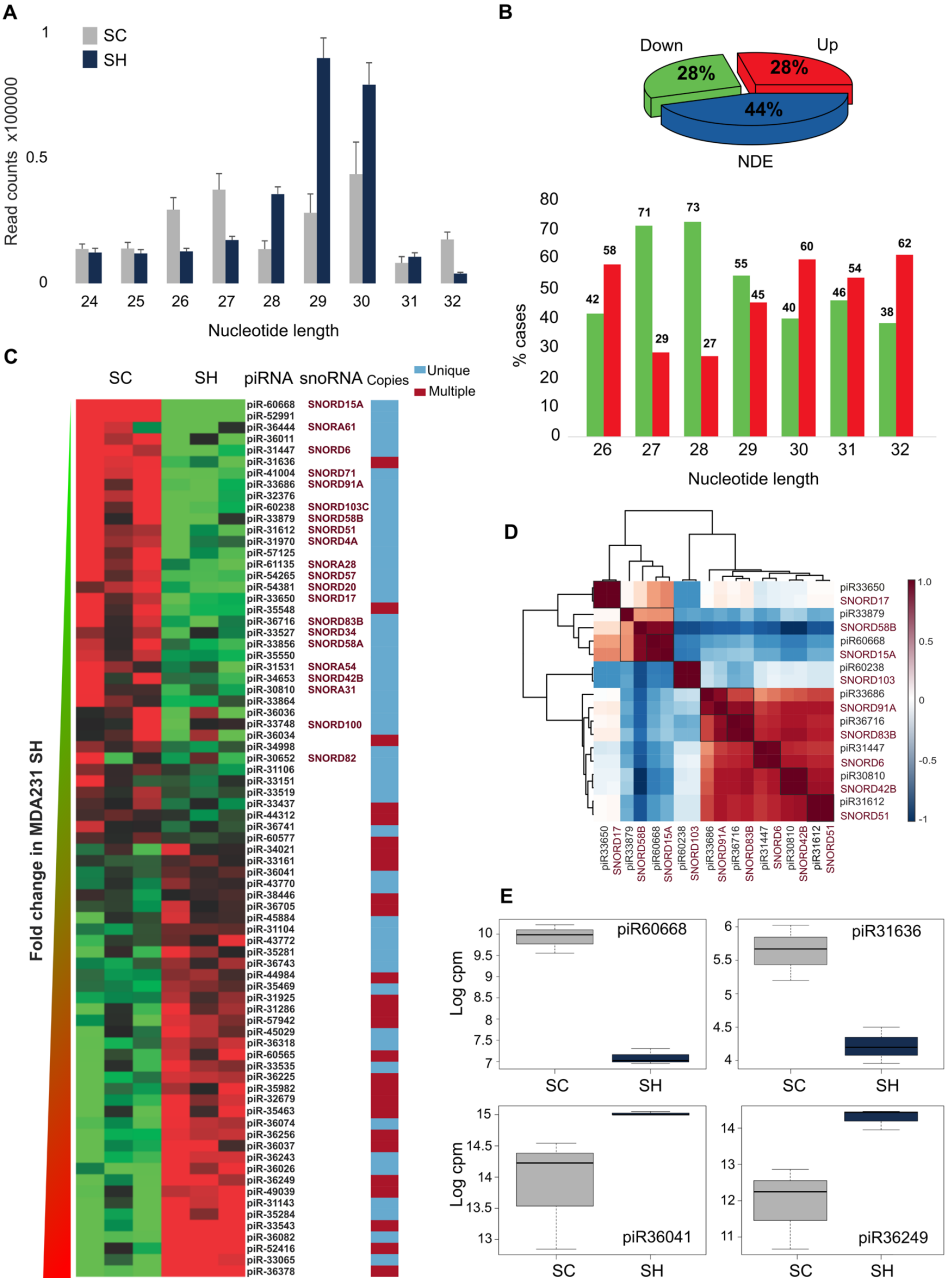
piRNAs **(A)** Length distribution of reads assigned to piRNAs in SC and SH libraries. **(B)** Piechart representation of the percentages of differentially expressed piRNAs and barchart of the frequencies of piRNAs in the upregulated and downregulated groups distributed according to their nucleotide length. **(C)** Heatmap representation of the differentially expressed piRNAs; the name of the host snoRNAs when it corresponds, and copies in the genome are indicated. **(D)** Corrplot of the pairs piRNA-snoRNA. **(E)** Boxplots of four representative piRNAs differentially expressed. NDE: Non-Differentially Expressed.

Although total piRNAs abundance did not change after GPAT2 silencing, an upper shift in read length distribution was observed (Figure 2A). In SC cells, length distribution was bimodal, with peaks at 27 and 30 nt, whereas in SH cells, only one peak at 29 nt was obtained.

Differential expression analysis revealed that of the 137 piRNAs identified in SC cells, 77 (56%) were differentially expressed after GPAT2 silencing (p≤0.05, FC≥|1.5|), with 38 upregulated (28%) and 39 downregulated (28%) (Figure 2B, Supplementary Table 2).

Genomic features of deregulated piRNAs were annotated according to their sequences based on curated databases (piRNA bank, NCBI_Nucleotide and UCSC genome Browser). Length distribution in both groups indicated that piRNAs of 27 and 28 nt in length were significantly associated with the downregulated group (p value ≤0.05). There were no such differences in the other lengths (Figure 2B). Interestingly, most of the downregulated piRNAs (32/39, 82%) are single copy (p value≤0.05), being mainly intragenic (27/32, 84%); whereas in the upregulated group, piRNAs with single (18/38, 47%) and multiple (20/38, 52%) copies showed similar frequencies (p value≤0.05) (Figure 2C, Supplementary Table 2); however, single copy upregulated piRNAs were mostly intergenic (14/18, 77%) (p value ≤0.05). Strikingly, snoRNAs constituted the host gene of 22 out of 27 (81%) intragenic single copy downregulated piRNAs, which is 56% of all downregulated piRNAs, with a probability value ≤0.05 when compared with the upregulated piRNAs. Moreover, piR-36011, a multiple copy downregulated piRNA, maps to the loci of the SNar genes (small NF90-associated RNAs). By constrast, none of the upregulated piRNAs is hosted in a SNOR or SNAR gene.

Considering that it has been proposed that certain piRNAs are derived from snoRNAs precursors [15], and that piRNAs are tissue restricted, we evaluated whether there is a correlation in tissue distribution among the downregulated piRNAs and their hosted snoRNAs. Using DASHR database we analyzed the tissue profile of the piRNAs and the host snoRNAs that were available in the database. Unsupervised clustering based on Pearson correlation was assayed on the nine pairs of piRNA-snoRNA obtained from the search. In all cases an almost prefect correlation (~1) was observed, coincident with a co-expression pattern (Figure 2D).

Interestingly, four of the top-five upregulated piRNAs previously identified in breast cancer growing cells [10], were found downregulated in the SH cells (piR-31636, piR-57125, piR-35548 and piR-57125). Similarly, piR-36041 and piR-43772 which were markedly downregulated in MCF7 growing cells, were found upregulated in the SH cells. Furthermore, of the latter group, piR-36743, piR-36318 and piR-36249 were previously found underexpressed in BC tissues compared to their normal counterparts [10]. All these data agree with the less proliferative phenotype of the SH cells. Expression of four representative piRNAs is displayed in Figure 2E.

Based on published evidences that piRNAs would be involved in mRNA target repression via imperfect base-pairing between the piRNA and the potential target [16], we searched for putative mRNA targets by base complementarity for all the differentially expressed piRNAs. We considered the percentage of complementarity, the strand sense, the mRNA region mapped (5’UTR or 3’UTR), the total score provided by the NCBI Nucleotide Blast, the conservation among species, and not only mRNAs but also pseudogene transcripts and long ncRNAs (Supplementary Table 3). The number of targets varied considerably for each piRNA, ranging from no-hits to hundreds of mRNAs. After filtering, based on the mentioned criteria, we obtained a reduced list of targets (Supplementary Table 3). The functional enrichment of piRNA targets yielded terms mainly linked to lipid metabolism that included *sphingolipid de novo biosynthesis*, *peroxisomal lipid metabolism* and *synthesis and interconversion of nucleotide di- and triphosphates*, among others (Supplementary Table 3). The expression of the putative piRNA target ACSS3, a gene coding for acyl-CoA synthetase short-chain family member 3, was assessed by qPCR; as expected, ACSS3 gene expression decreased 90% in SH cells (Supplementary Figure 3).

### GPAT2 silencing upregulates specific tRF that might derive from tRNAs associated to a proliferative cellular status

**Figure 3:**
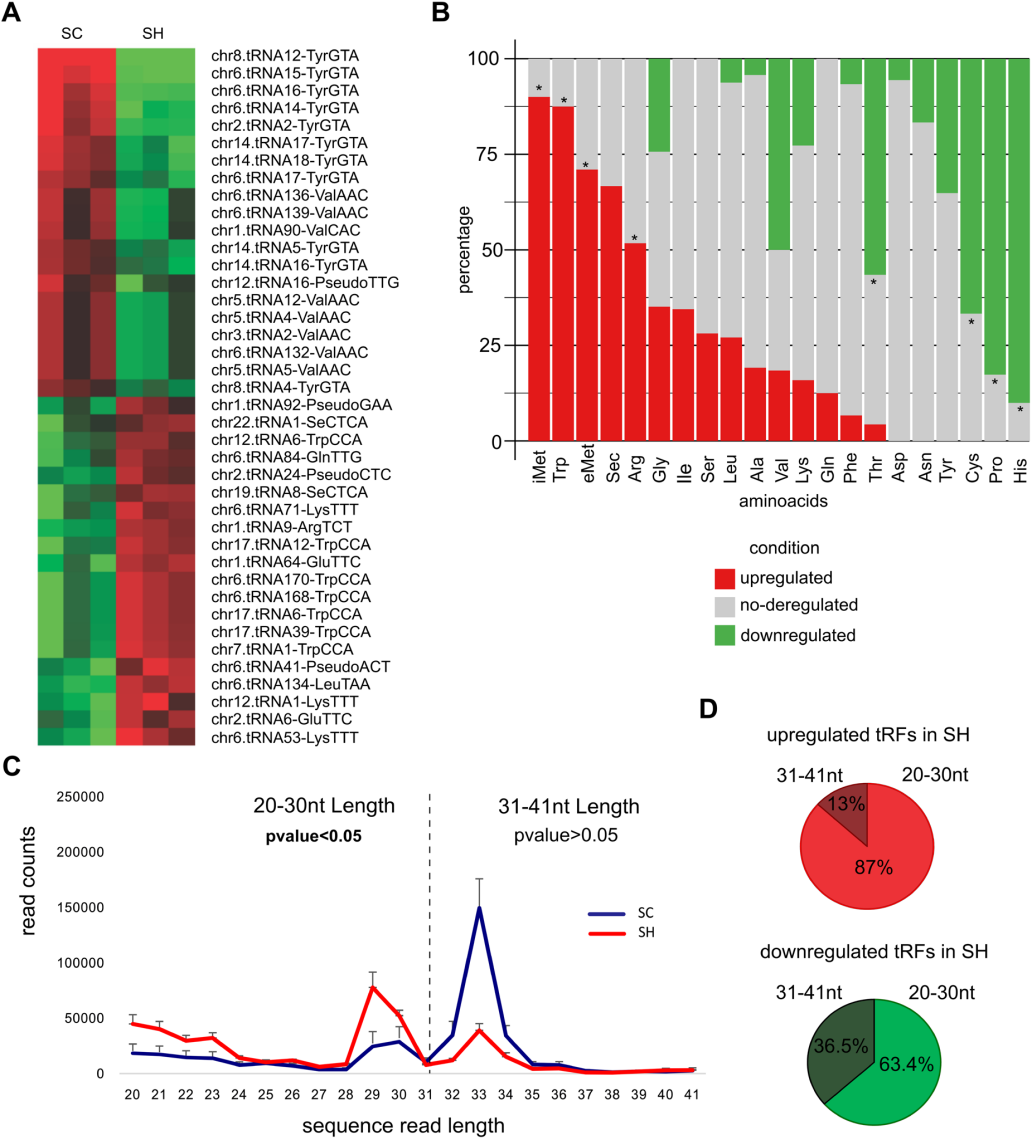
tRF **(A)** Heatmap representation of the top 40 deregulated tRF identified in the comparison SC *vs* SH cells and annotated according to the name of the mature tRNA. **(B)** Stacked barplot of the frequency distribution of tRF considered according to the amino acid they carry. Only the name of the amino acid is indicated. The asterisk indicates significantly overrepresented (tRF)-amino acids in the up and downregulated groups. **(C)** Distribution of reads assigned to tRF based on their nucleotide length. **(D)** Piecharts of the percentages of tRF classified according to their nucleotide length.

It has been established that tRF can be classified into two groups: “tRNA halves”, that are 30-35 nt in length and belongs to the single cleavage of the mature tRNA at its anticodon loop, and tRF that are derived from mature or precursor tRNAs after cleavage at either the D-loop or the T-loop, giving 5′-tRF and 3′-tRF [17]. Although total reads assigned to tRF did not change after GPAT2 silencing, 275 tRF were identified as differentially expressed (FC≥|1.5|, pvalue≤0.05), with 147 tRF downregulated and 128 tRF upregulated (Supplementary Table 4). Figure 3A shows the top 40 deregulated tRF annotated according to the corresponding mature tRNA ID. We then evaluated the frequency distribution of each tRF species in the upregulated and downregulated groups, compared with the total tRNA genes for each species in the genome (GtRNAdb). Results indicated that downregulated tRF are derived mainly from His-tRNA (9 out of 10, 90%), Pro-tRNA (19 out of 23, 83%), Cys-tRNA (26 out of 39, 67%) and Thr-tRNA (13 out of 23, 56.1%), whereas upregulated tRF are derived mainly from iMet-tRNA (9 out of 10, 90%), eMet-tRNA (7 out of 10, 70%), Trp-tRNA (7 out of 8, 87.5%) and Arg-tRNA (Figure 3B, Supplementary Table 5). When evaluating the length distribution of reads assigned to tRF a shift in length was observed in SH cells; after GPAT2 silencing, the number of longer tRF (31-41 nt) decreased whereas the number of those with smaller length (20nt-30nt) significantly increased (Figure 3C). This means that smaller tRF (20-30 nt) were more abundant in the upregulated group whereas larger tRF (31-40 nt) were more restricted to the downregulated group (Figure 3D).

**Figure 4:**
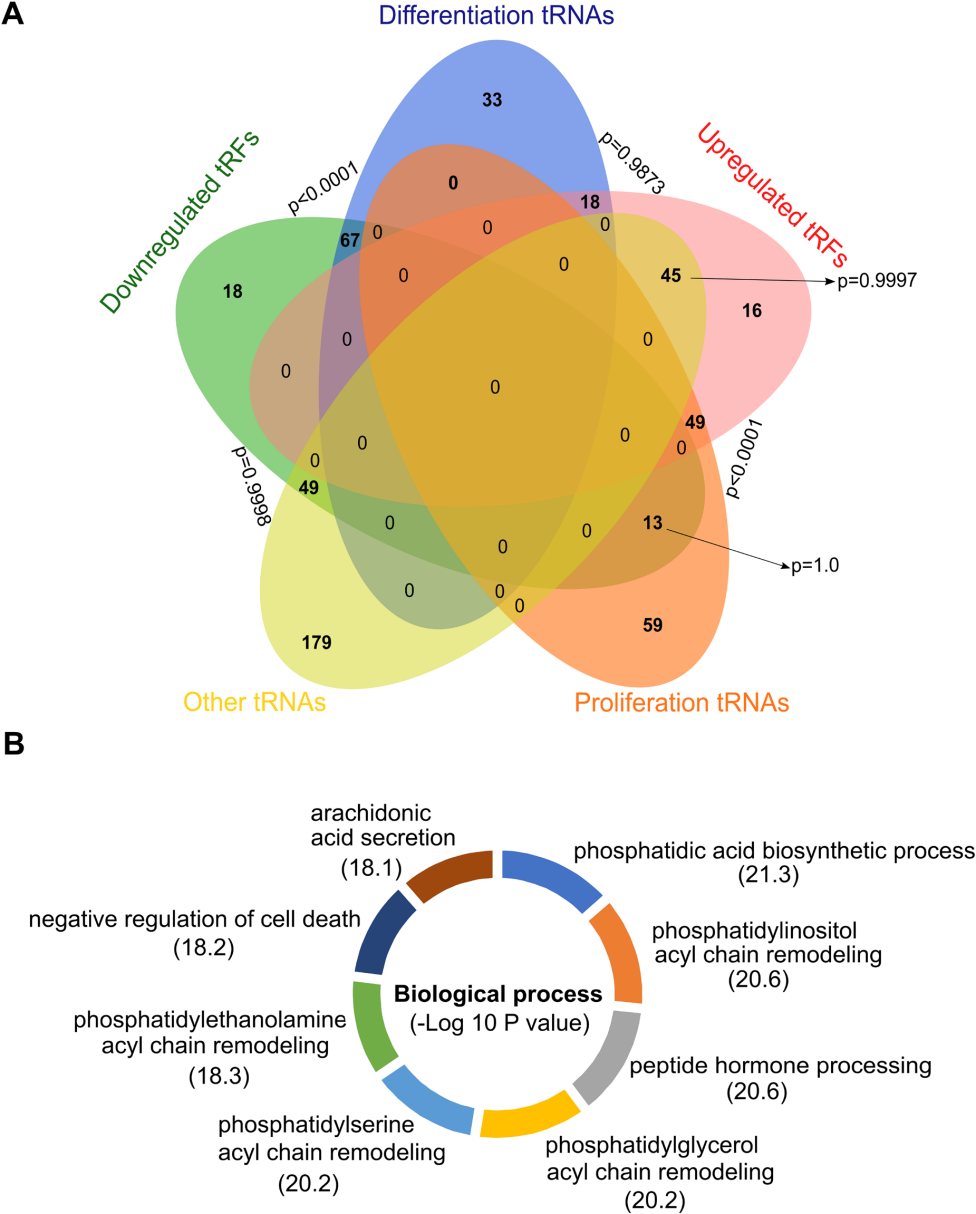
tRF and its associated tRNAs **(A)** Euler diagram of the comparison between the upregulated and downregulated tRF with the Gingold classification of tRNAs. **(B)** Functional enrichment of the putative proteins obtained from the (tRF)-amino acid frequencies.

To find a biological meaning for deregulated tRF, we used the classification for tRNAs previously proposed by Gingold et al [18]. The authors established the existence of two distinct translational programs that operate during proliferation and differentiation, which eventually coordinate the supply and demand of tRNAs. Differentiated cells are less proliferative, and proliferating cells are typically not terminally differentiated, hence, according to the cellular status at which they are expressed, Gingold et al. classified the tRNAs into *proliferation* and *differentiation* tRNAs. Using Euler diagrams, we observed a significant association (pvalue≤0.0001) between the subset *differentiation tRNAs* with the downregulated tRF in our analysis, whereas the opposite occurred with the upregulated ones, with a strong association (pvalue≤0.0001) to the *proliferation* tRNAs subset (Figure 4A, Supplementary Table 6).

Because tRNAs are the supply of amino acids to build proteins, we asked whether from the frequency of upregulated and downregulated tRF we could establish a protein profile with the aim of having a different approximation about which biological processes are being affected by GPAT2 silencing. We used the abundance of each species of tRF and defined its frequency based on the amino acid they carry (Supplementary Table 7). We then used the CompSite expasy database and obtained a list of scored putative proteins (Supplementary Table 7). Interestingly, functional enrichment of these proteins enabled us to identify the biological processes previously associated to GPAT2, such as *phosphatidic acid biosynthesis*, *phospholipid acyl chain remodeling* and *regulation of cell death*, among others (Figure 4B).

### GPAT2 silencing affects the expression of miRNAs targeting to genes related to cell growth and lipid metabolism

**Figure 5:**
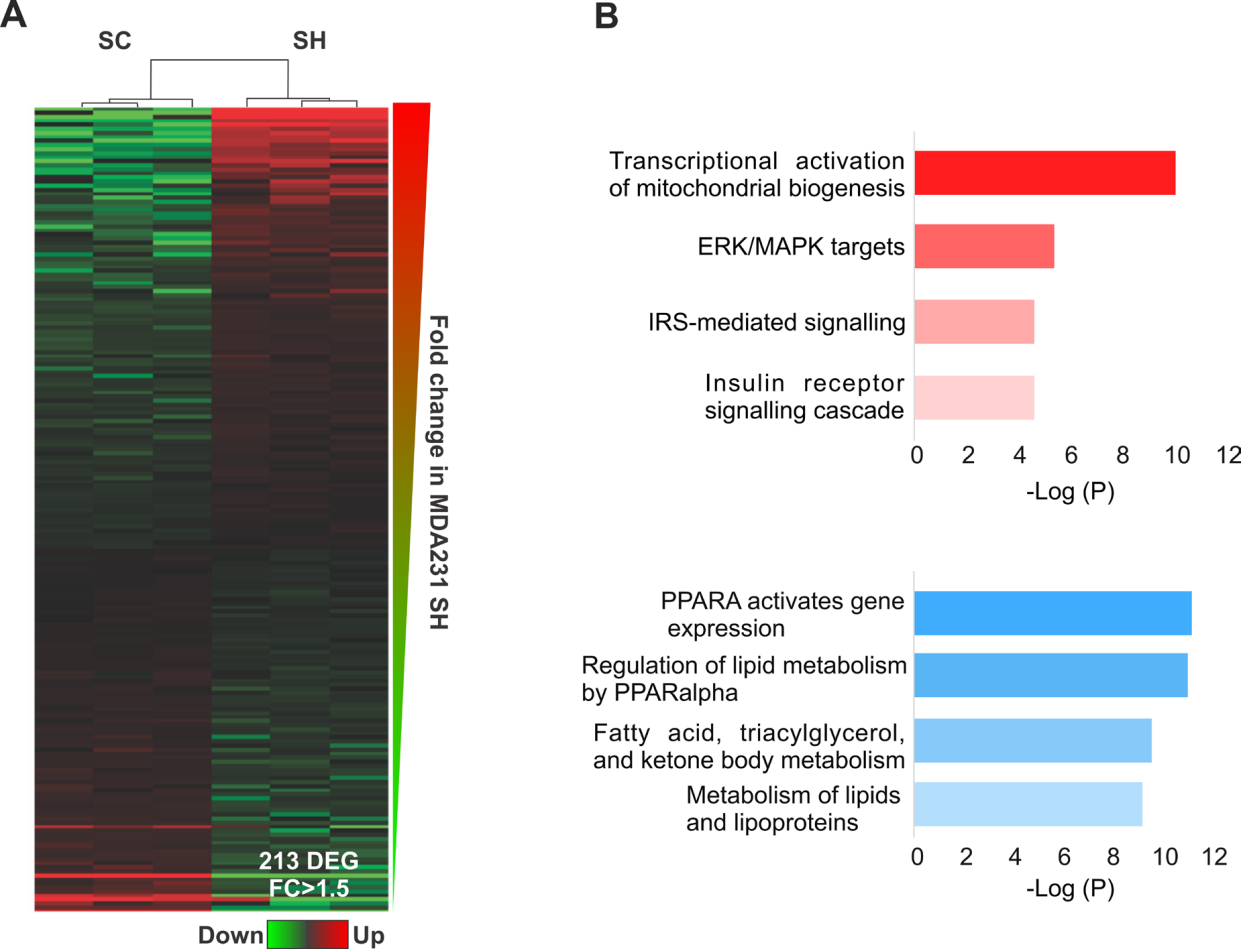
miRNAs **(A)** Heatmap representation of deregulated miRNAs in SC *vs* SH cells. **(B)** Functional enrichment of the targets of upregulated miRNAs (red) and downregulated miRNAs (light blue).

In contrast to piRNAs and tRF, miRNAs abundance significantly decreased after GPAT2 silencing (Figure 1, Supplementary Table 8). Unsupervised hierarchical clustering analysis of differentially expressed miRNAs demonstrated a clear segregation of SC and SH cells (Figure 5A). Statistical analysis revealed 213 transcripts differentially expressed (109 upregulated & 104 downregulated) between the two cell line conditions (Supplementary Table 8). We choose miR-5100 and miR-34 to validate small RNAseq data. Semiquantitative RT-PCR experiment demonstrate that, as expected, pre-miR-5100 was upregulated whereas pre-miR-34 was downregulated in SH cells (Supplementary Figure 4).

To predict putative targets, miRDB database was used and the 50 best ranked putative targets for each deregulated miRNA were selected (Supplementary Table 9). By pivot tables (cross tabulations), the more relevant targets present in at least 5 miRNAs (>5%) were extracted (Supplementary Table 9). This means to select the genes that constitute targets for more than five miRNAs. Following this criterion, two lists of putative gene targets were obtained, one of 51 genes for the upregulated miRNAs, and the other of 109 genes for the downregulated miRNAs (Supplementary Table 9).

To identify biological processes associated to miRNA targets, functional enrichment analysis using ENRICHR database was performed. Pathways analysis revealed specific terms associated to *mitochondrial biogenesis and IGF1R signaling* for genes associated to upregulated miRNAs, and *oxidative metabolism of lipids and lipoproteins* for genes associated to downregulated miRNAs (Figure 5B). Among the putative genes targeted by the upregulated miRNAs were APPL1 and SPRED1, both playing critical roles in cell proliferation [19, 20]. The expression of these genes was assessed by qPCR and as expected, both were found downregulated in SH cells (Supplementary Figure 3).

### GPAT2 modulates miRNAs associated with poor prognosis in breast cancer

**Figure 6:**
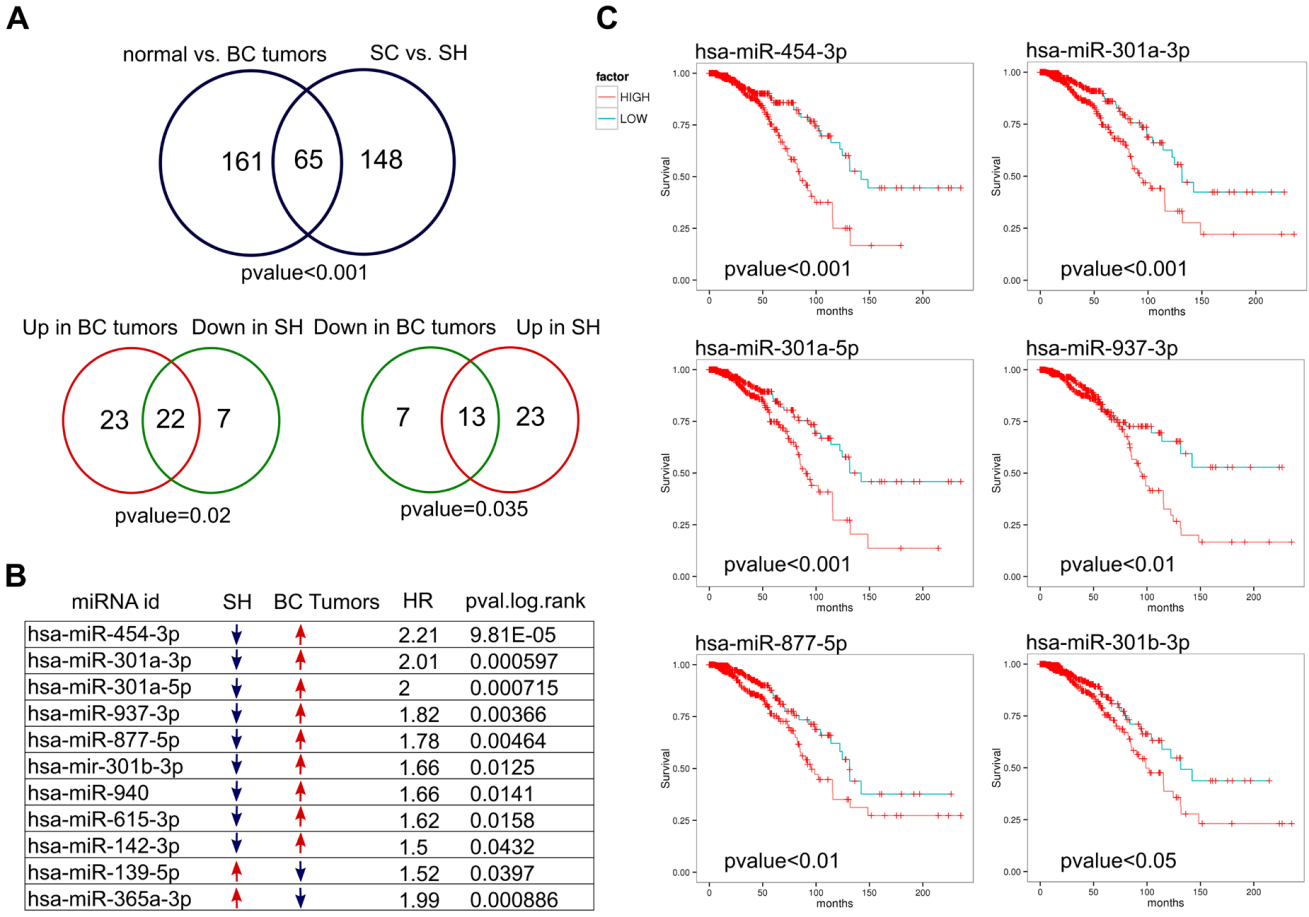
Deregulated miRNAs and breast cancer tumors **(A)** Comparison of differentially expressed miRNAs in SC *vs* SH cells with differentially expressed miRNAs in normal *vs* breast cancer tumors indicates a significant association. Venn diagrams of opposite groups (Up *vs* Down) also showed a significant association **(B)** Nine of the 22 miRNA downregulated in SH cells and activated in breast cancer are associated with poor prognosis in breast cancer. Figure **(C)** shows the Kaplan Meier curves of 6 of them. BC: breast cancer. HR: Hazard ratio.

To determine whether deregulated miRNAs could have an impact on the survival of patients with breast cancer (BC), we performed an analysis using the YM500 database. According to YM500 there are 226 miRNAs differentially expressed between BC tumors (n=994) and normal breast (n=103) (Supplementary Table 10). We compared this group with the 213 deregulated miRNAs identified in our study. We used the normal approximation to the binomial distribution as previously described [21] to calculate whether the number of deregulated miRNAs derived from each cross-platform comparison was of statistical significance. We found sixty-five miRNAs common to both groups (pvalue≤0.05, Figure 6A). Of the 65 miRNAs, 45 are upregulated and 20 downregulated in YM500 BC tumors, while 36 and 29 are upregulated and downregulated, respectively, in the SH cells from our analysis. Interestingly, we found a significant association between the miRNAs upregulated in breast cancer tumors with the miRNAs downregulated in the SH cells (22 miRNAs in common, pvalue≤0.05), as well as between the miRNAs downregulated in BC tumors and the upregulated in the SH cells (13 miRNAs in common, pvalue≤0.05, Figure 6A). We then found that 9 of the 22 miRNAs that are downregulated in the SH cells have a significant impact on breast cancer patient survival if they are upregulated in tumors; whereas only 2 of the 13 upregulated miRNAs in the SH cells showed poor prognosis (Figure 6B). Figure 6 C shows the Kaplan Meier curves of 6 of the 9 miRNAs downregulated after GPAT2 silencing and upregulated in breast cancer tumors. Moreover, considering that MDA-MB-231 cells are negative for hormone receptors, we performed the survival analysis on a defined group of ER- and PR- breast cancer tumors (n=218) for each of the significant miRNAs identified in the comparison normal vs tumor, but no significant association with overall survival was found in any of the miRNA analyzed.

## DISCUSSION

In this study, we described how the landscape of sncRNAs is affected by GPAT2 silencing in triple-negative breast cancer MDA-MB-231 cells, which normally express GPAT2. Based on the results obtained by Shiromoto et al. [4], demonstrating that GPAT2 participates in piRNA biogenesis in mouse germline stem cells, we hypothesized that this gene could also be involved in piRNA metabolism in somatic MDA-MB-231 cells, where piRNA synthesis was proved to be active [10]. By shRNA-mediated gene silencing we showed that although GPAT2 knockdown did not change significantly the total amounts of piRNAs, a shift in small RNA read length distribution was observed and specific piRNAs were deregulated. The most relevant finding lies in the group of downregulated piRNAs, whose genomic characteristics are homogeneous and clearly distinguishable from the upregulated group. First, 82% of them have a single copy in the human genome, of which, 81% - with 100% identity- are in the body of snoRNA genes. Second, we found a high tissue-specific correlation between the piRNA-snoRNA pairs, suggesting the co-expression of both RNAs. These results refer to the mechanism of the primary biogenesis of piRNAs, in which piRNAs precursors are transcribed from piRNAs clusters, and then processed into piRNA intermediates, which subsequently are trimmed and modified by methylation to led to mature piRNAs [22]. Considering that snoRNA genes are 65-300 nt long, it is possible to speculate that they may be precursors or intermediates in the production of certain class of piRNAs, and that GPAT2 is directly involved in this process. In this respect, it has been shown that certain piRNAs are derived from snoRNAs [15], and that these play specific roles in transcriptional and post-transcriptional regulation of gene expression [23, 24]. It is also worth mentioning that among the downregulated piRNAs it was found piR-36011. This piRNA is encoded in multiple sites in the genome and each copy maps in one of the 14 copies of the small NF90- associated RNA A genes (SNAR-A1 to 14). SnaRs are transcribed by RNA polymerase III and display restricted tissue distribution, with high expression in normal testis and discrete areas of the brain, and in many immortalized human cell lines compared to their pre-immortal counterpart [25]. Moreover, snaR genes are predominantly located in three clusters on chromosome 19 and have been duplicated as part of a larger genetic element. Like snoRNA derived piRNAs, piR-36011 could be originated from the processing of a precursor or intermediate SNAR.

We also searched for potential targets of the deregulated piRNAs, based on sequence complementarity. According to the parameters of the E-value, nucleotide match, mismatches, score and the orientation of the targeting strand, we identified a few potential target genes. Functional enrichment analysis revealed that the products of these RNAs are involved in lipid metabolism.

Another relevant finding is that deregulated piRNAs correlate with the less tumorigenic SH phenotype. For instance, many of the downregulated piRNAs (piR-31636, piR-57125, piR-35548 and piR-57125) were previously found upregulated in breast cancer growing cells and/or in breast tumors compared to their normal tissues, whereas many of the upregulated piRNAs were previously associated to a breast cancer growth arrested phenotype (piR-36743, piR-36318, piR-36249, piR-43772, piR-36041) [10].

In previous studies we have shown that GPAT2 promotes cell proliferation and its expression is inversely correlated with cell differentiation status in cancerous and in 3T3L1 fibroblast cells [3, 6]. In this work, we observed a significant association between the subset ‘*differentiation tRNAs*’ [18] and the downregulated tRF identified in our analysis, whereas the opposite occurred with the upregulated ones, with a strong association to the *proliferation* tRNAs subset. Under the assumptions that cellular tRNA pool constitutes a relevant prime factor that controls translation, and that variations in the expression of a given tRNA would affect the translation of all genes that need such tRNA, we speculate that the increase of tRF after GPAT2 silencing would be associated to a decay of specific tRNAs, affecting the synthesis of specific proteins. Therefore, we used the deregulated tRF -considered as products of tRNA degradation- to establish a putative profile of affected proteins. Interestingly, we identified proteins related to phospholipid biosynthesis and cell growth, two major processes previously linked with GPAT2.

miRNAs are the most studied ncRNAs in the context of cancer biology. In their processing, they markedly differ from piRNAs and tRNAs. Of the three classes of sncRNAs analyzed in this study, only miRNAs showed a significant variation in the total abundance of aligned reads, with a decrease in the SH cells, suggesting an impact in the overall production of miRNAs. Using bioinformatics analysis, we identify a set of potential targets for the upregulated and downregulated miRNAs. Target genes of miRNAs would be associated with processes linked to lipid biosynthesis, cell growth and proliferation.

To evaluate if the deregulated miRNAs in the MDA-MB-231 cells might have a role in breast cancer, we used the YM500 database, which contains >8000 small RNA sequencing data sets, and integrated analysis results for various cancers miRNome studies. We found a significant overlap between the miRNAs differentially expressed in the comparison normal breast *vs* breast cancer obtained from YM500 database, with those found affected by GPAT2 silencing in the present study. We also identified 9 miRNAs downregulated by GPAT2 silencing that are usually upregulated in breast cancer and are associated with a worse survival prognosis. Therefore, differentially expressed miRNAs identified here in SC *vs* SH cells show a similar pattern in normal *vs* breast cancer. On the other hand, within a cohort of PR- and ER- tumors miRNA expression was not correlated with overall survival. We speculated that there might be different reasons. First, the reduced number of cases with hormone receptor negative status and follow up data. Second, although there have been defined different molecular subtypes of hormone receptor negative tumors, particularly triple negative breast cancer tumors, they usually constitute a discrete breast cancer subgroup with a homogenous behavior in respect of the prognosis and overall survival. Despite this, we conducted an exhaustive search in the literature for each of the miRNAs highly correlated with survival in this study. It has been shown that miR-454 is associated with poor prognosis in triple negative breast cancer tumors [26]; similarly, miR-301 mediates cell proliferation in invasive breast cancers [27]. Overall, our data demonstrate that beyond the molecular subtype of the cell line employed, some of the miRNAs identified in our model could be powerful prognostic markers in breast cancer, as was postulated in other studies, and some of them could constitute new ones to further validate in future studies.

The specific characteristics of deregulated piRNAs, tRF and miRNAs strongly correlate with processes associated with GPAT2 in previous studies, indicating a specific cause-effect of GPAT2 silencing. The mechanisms by which GPAT2 deregulate the expression of small non-coding RNAs remains unknown, but in this study, we show that GPAT2 modifies the abundance and length of specific piRNAs, tRF and miRNAs. The involvement of outer mitochondrial membrane proteins in primary piRNA processing was previously described [28, 29, 30]. It was demonstrated that a member of the Drosophila glycerol-3phosphate acyl-transferase (GPAT) family proteins, named Minotaur, collaborates with Zucchini in primary piRNA processing near the mitochondrial membrane [31]. This protein is homologous to the mouse GPAT2, and its role in piRNA metabolism is independent of its acyltransferase motif. In this sense, we and others have previously postulated that GPAT2 protein contains intrinsically disordered regions [32, 33]; hence it is possible to speculate that GPAT2 could act as a scaffold protein to function in the processing of specific small ncRNAs that eventually control lipid biosynthesis and cell proliferation.

## MATERIALS AND METHODS

### Cell lines and culture conditions

The human MDA-MB-231 cell line, derived from mammary adenocarcinoma, was purchased from ATCC and maintained in DMEM supplemented with 10% FBS, 100 U/ml penicillin, 100 mg/ml streptomycin and 2 mM glutamine. Cells were grown at 37°C in a 5% CO2 atmosphere with 98% relative humidity.

### GPAT2 silencing

For human GPAT2 silencing, MDA-MB-231 cells were transfected using Lipofectamine 2000 Reagent (Life Technologies) with HuSH-29 plasmid (OriGene) coding for shRNA against human GPAT2 mRNA and selected for puromycin resistance to generate the respective MDA-MB-231 silenced cell line (SH). A non-effective scrambled sequence shRNA plasmid was used to create a negative control (SC). Both plasmids also contain a sequence coding for green fluorescent protein driven by a CMV promoter.

### Small RNA sequencing

Total RNA was extracted from the cell line using the standard RNA extraction method with QIAIzol (Qiagen), quantitated with NanoDrop-1000 spectrophotometer (Thermo Fisher Scientific) before integrity assessment with an Agilent 2100 Bioanalyzer (Agilent Technologies). For small RNA-seq, 1 μg of total RNA from SH and SC cells was used for library preparation with Illumina TruSeq small RNA sample preparation Kit. Three independent experiments (two clones per cell line) for each condition, were sequenced (10 pM) on HiSeq2500 (Illumina) with single read for 51 cycles. Small RNA sequencing data was analyzed using iSmaRT [34] to identify the sncRNA families studied, i.e. miRNAs (miRBase v21), PIWI-interacting RNAs (piRNABank), and tRNA-derived fragments (tRF, Human genome assembly, GRCh37/hg19) with Minimum Read Count of 3. Rfam and RefGene correspond to reads mapped to Rfam [35] and Refgene (known human protein-coding and non-protein-coding genes) databases.

### Bioinformatics analysis

To identify differentially expressed miRNAs, piRNAs or tRF between SH and SC samples, we utilized the DESeq2 algorithm based on the normalized number of counts mapped to each sncRNA transcript [36]. Functional enrichment analyses were performed using the databases DAVID, http://david.abcc.ncifcrf.gov/), Enrichr (http://amp.pharm.mssm.edu/Enrichr/) and FunRich (www.funrich.org), based on the list of genes associated to the deregulated sncRNAs (P-adj. ≤0.05; FC ≥|1.5|). Data integration, heatmap visualization of differentially expressed transcripts and functional enrichment plots were done with R/Bioconductor packages and the Multi Experiment Viewer software (MeV v4.9) [37]. To validate the bioinformatic analysis of small RNA-seq experiments, we compared the global miRNA expression profile of SC from our study with the global miRNA expression profile of the MDA-MB-231 and MCF10 cell lines obtained from the study of Zhou et al., 2014 [14], in which the authors profiled the cellular small RNAs isolated from these two cell lines by Solexa deep sequencing. Briefly, normalized data were downloaded from GEO (ID#GSE50429) and the miRNAs in common to our libraries were selected (n=228). The comparison was made using a linear regression model in R.

The name or GenBank ID, chromosome number, genomic position, strand orientation and sequence length of piRNAs was obtained from piRNAbank (http://pirnabank.ibab.ac.in/simple_search.html), and validated with the NCBI Nucleotoide Database (https://www.ncbi.nlm.nih.gov/nuccore/). The number of copies in the genome and the genomic loci was obtained from the UCSC Genome Browser. To identify potential target genes of relevant piRNAs, we employed the NCBI database (Human Genomic plus Transcript) based on sequence complementarity using the reverse complement of the piRNA sequence as input. The HomoloGene tool from the NCBI database was employed to evaluate the grade of conservation of the selected putative mRNA targets among different mammalian species. For miRNA target prediction and functional annotations, we used the miRDB online resource (http://www.mirdb.org/miRDB/). To evaluate differences in the abundance of each species of tRF among the upregulated and downregulated group, we used Fisher Test to compare their frequencies with the expected frequencies according to the Genomic tRNA database (http://gtrnadb.ucsc.edu/). For the identification of putative proteins based on amino acids composition, we employed the AAcompIdent tool (http://web.expasy.org/aacompident/). For piRNA and snoRNA expression levels across human tissues and cell lines we employed the DASHR database (http://lisanwanglab.org/DASHR/smdb.php).

To evaluate and compare differentially expressed miRNAs found in this study with miRNAs deregulated in breast cancer tumors, we used the YM500v3 database (http://driverdb.tms.cmu.edu.tw/ym500v3) which employ TCGA data to contrast normal *vs* cancer tissue. We selected the comparison of 1096 primary solid breast cancer tumors against 104 samples of normal breast tissue [38]. Survival section of YM500 database was employed to survival analysis of common deregulated miRNAs.

### qPCR and semiquantitative RT-PCR experiments

Total RNA was isolated from SC and SH cell lines using QIAzol (Qiagen). 1 μg RNA was used for cDNA synthesis employing High Capacity Reverse Transcription Kit (Applied Biosystems). A cDNA dilution (1/10) was used for the qPCR reactions with IQ Sybr Green Super Mix (Bio-Rad). Primers were designed to amplify GPAT2 (forward: ATCCTACTGCTGCTGCACCT; reverse: ACAGCAGCTTTGCACTCAGA), ACSS3 (forward: CGTCCCAGGATACAATGTTATGAT; reverse AAAAAGCCCCAGGTGGCAAT), APPL1 (forward: AGCGGGAGAAGTGAAAGTAAT; reverse: GGCTACTGCTAAGGACAACAA), SPRED1 (forward: TCTCAAGGATGCCCCGAATC; reverse: GGCTCACTGGTAACAACTGTCT), and TBP (forward: TATAATCCCAAGCGGTTTG; reverse GCTGGAAAACCCAACTTCT). An AriaMx Real-Time PCR System (Agilent Technologies) was used for measurements. RNA expression was quantified in triplicate using the ΔCt method and normalized to TBP (TATA binding protein) expression level using Qbase software.

To assess pre-miRNA expression by semiquantitative RT-PCR, 500 ng of total RNA was used for cDNA synthesis employing High Capacity Reverse Transcription Kit (Applied Biosystems) and used to amplify with primers for pre-miR-34a (forward: TGTTTCTTTGGCAGTGTCTTAGC; reverse: TGCAGCACTTCTAGGGCAGTAT), pre-miR-5100 (forward: CATGAGGAGCTGGCAGTGG; reverse: GTCCTGTGGCCAGTTAGAGG) and TBP (forward: TATAATCCCAAGCGGTTTG; reverse GCTGGAAAACCCAACTTCT), using a Veriti 96-well Thermal Cycler (Applied Biosystems) with the following cycling program: 0:30 min at 98°C; 35 cycles of 0:10 min 98°C, 0:40 min at 58°C and 2:00 min at 72°C, and a final hold of 7 min at 72°C. Amplification products were run on Ethidium Bromide stained 1.5% agarose gel along with a 1 kbp DNA ladder (Life Technologies) to confirm the expected molecular weight of the amplification products. Quantification of the band intensities was performed by Image J program normalizing the intensity value to that of TBP.

### Western blot

One-hundred μg of total protein from SC and SH cells was separated on 10% SDS-PAGE, transferred to a polyvinylidene difluoride membrane (BioRad) and probed with 1/1000 anti-GPAT2 antibody (Sigma HPA036841). Anti-β-actin antibody (Abcam ab8227) at a dilution 1:2500 was used as a gel-loading control. Membranes were then washed extensively and probed with horseradish peroxidase-conjugated goat anti-rabbit or anti-mouse IgG antibody (Thermo-Pierce). For chemiluminescent detection, membranes were incubated with Super Signal detection kit (Thermo-Pierce).

## SUPPLEMENTARY MATERIALS FIGURES AND TABLES




