# Supplementary material for: Small non-coding RNA landscape is modified by GPAT2 silencing in MDA-MB-231 cells
Source: Oncotarget. 2018 Jun 15;9(46):28141–54. doi: 10.18632/oncotarget.25582 (PMC6021339; doi:10.18632/oncotarget.25582)
Supplement: Supplementary file 1 [file oncotarget-09-28141-s001.pdf]

Small non-coding RNA landscape is modified by GPAT2 silencing in MDA-MB-231 cells

SUPPLEMENTARY MATERIALS

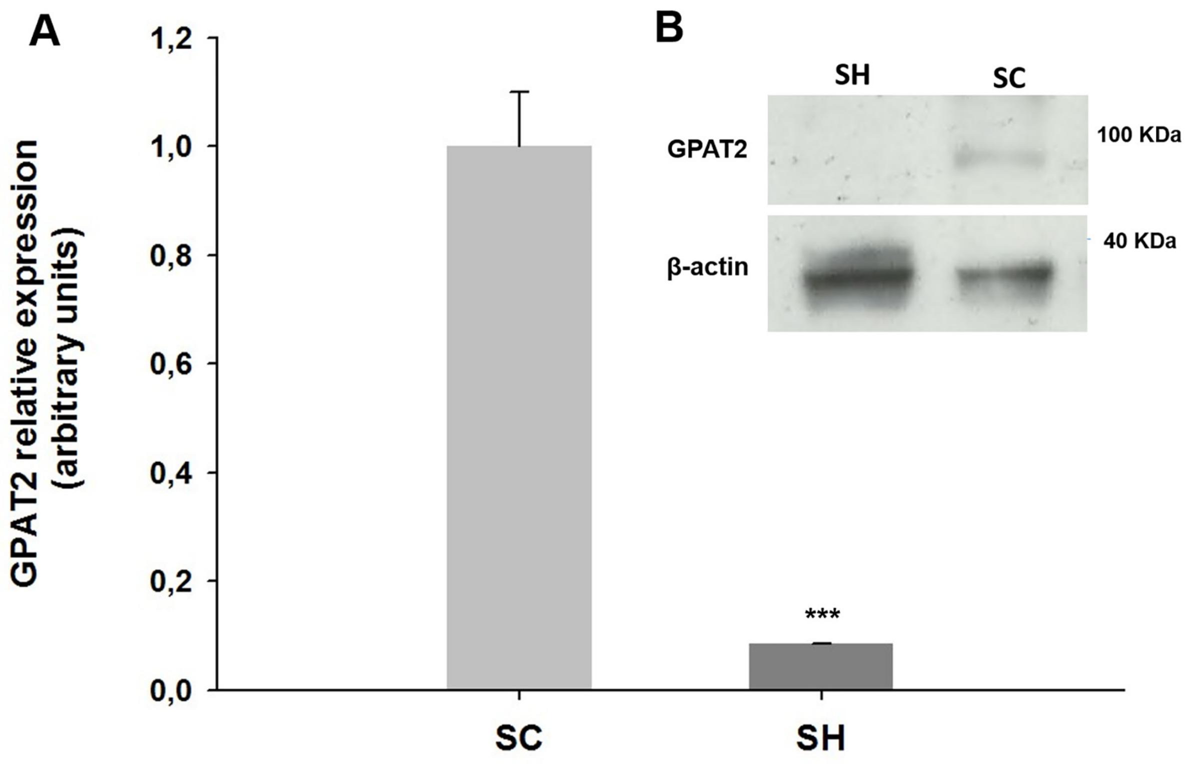

Supplementary Figure 1: GPAT2 knockdown in MDA-MB-231 cells. **(A)** GPAT2 mRNA expression was assessed by qPCR in SC and SH cells. **(B)** GPAT2 protein expression was analyzed by western blot in SC and SH cells using anti-GPAT2 and anti-βactin antibodies.

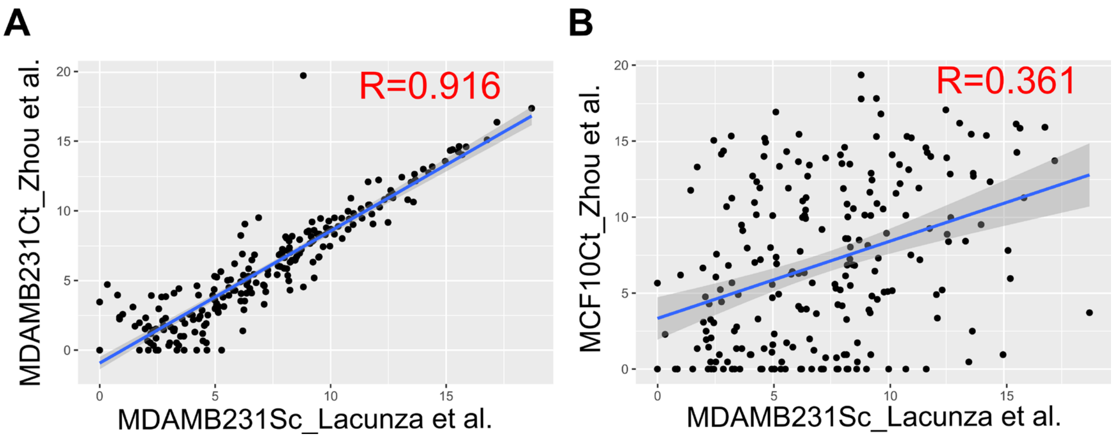

Supplementary Figure 2: miRNA expression profile. Comparisons among the miRNA expression profile obtained from SC cell line of the present study (Lacunza et al.) with those expression profiles obtained from MDAMB231 and MCF10 cell lines according to Zhou et al. (GEO ID#GSE50429). Dots represent individual miRNAs with their corresponding expression value. .

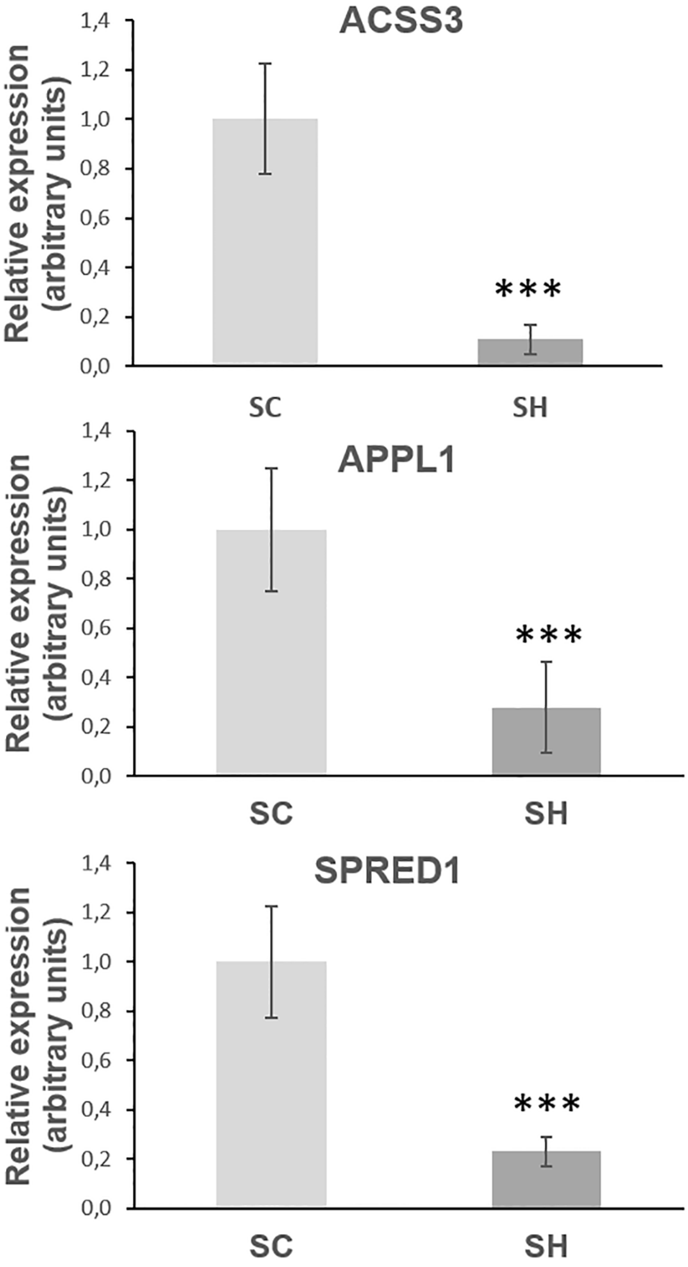

Supplementary Figure 3: piRNA and miRNA targets. Gene expression for ACSS3, a representative putative piRNA target, and for APPL1 and SPRED1, two representative putative miRNA targets, assessed by qPCR on SC and SH cells. In all cases, the expression level was normalized to that of TBP.

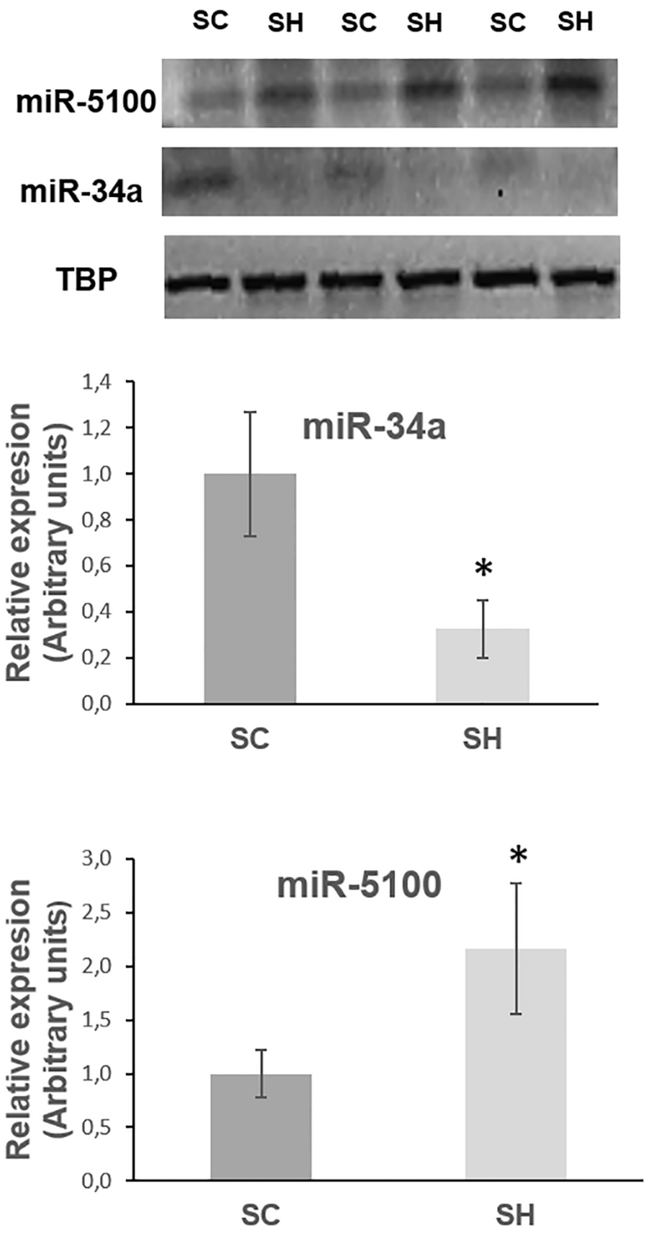

Supplementary Figure 4: miRNA expression in SC and SH cells. Gene expression level for representative miRNAs was assessed by RT-PCR on SC and SH cells. Expression level was normalized to that of TBP.

Supplementary Table 1: Frecuencies of small RNAs identifed by RNAseq in triplicates of MDAMB231SC and MDAMB231SH breast cancer cells

See Supplementary File 1

Supplementary Table 2: Total and differentially expressed piRNAs obtained by DESEQ2 analysis

See Supplementary File 1

Supplementary Table 3: Blast piRNAs; identification, functional enrichment and grade of conservation of potential mRNA targets

See Supplementary File 1

Supplementary Table 4: Total and differentially expressed tRFs obtained by DESEQ2 analysis

See Supplementary File 1

Supplementary Table 5: Distribution of frequencies of tRFs according to the amino acid they transport

See Supplementary File 1

Supplementary Table 6: tRNA analysis based on Gingold et al. classification

See Supplementary File 1

Supplementary Table 7: Protein prediction and Functional Enrichment based on Amino Acid Composition obtained from the differentially expressed tRNAs

See Supplementary File 1

Supplementary Table 8: Total and differentially expressed miRNAs identified by DESEQ2

See Supplementary File 1

Supplementary Table 9: Analysis of putative targets of differentially expressed miRNAs

See Supplementary File 1

Supplementary Table 10: Comparative analysis among differentially expressed miRNAs in SC-SH cells vs diff. expressed miRNAs in Normal-BC tumors

See Supplementary File 1
